# Transient response of bridge piers under eccentric impact of near-fault earthquake

**DOI:** 10.1038/s41598-022-21213-4

**Published:** 2022-10-05

**Authors:** Zihu Wang, Qingyan Zeng, Yantao Du, Wenjun An

**Affiliations:** 1 Modern Education Technology Center, Jiangxi University of Engineering, Xinyu, 338000 China; 2 School of Civil Engineering, Jiangxi University of Engineering, Xinyu, 338000 China

**Keywords:** Civil engineering, Mechanical engineering, Applied mathematics

## Abstract

To study the influence of near-fault earthquake on pier failure, establish a double-span continuous girder bridge. The seismic response of the bridge is calculated using the transient wave function expansion method and the indirect mode function superposition method. Solve the dynamic and displacement responses, and the effect of the vertical separation of the bridge on the eccentric compression of the pier is analyzed. The results show that under the near-fault vertical seismic action, the separation can significantly change the horizontal deformation at the top of the pier, and neglecting the separation may underestimate the eccentric compression of the pier. Calculations for different pier heights and girder spans show that separation has a greater effect on the longitudinal deformation of the pier top. Therefore, the reasonable design of longitudinal limit device on pier top in the near-fault area is helpful to reduce the damage of eccentric bridge impact.

## Introduction

Under the action of the earthquake, the damage of the bridge affects the rescue after the disaster and brings difficulties to the recovery after the disaster^1,2^. Given bridge collision, the current research focuses on the collision of adjacent beams and the collision between the main girder and abutment, and some achievements have been made^3^. Many scholars set up different models to calculate the collision force and set up a reasonable device to reduce the structural collision force^4–7^. However, few studies on vertical collision, the especially eccentric collision between the main girder and bridge pier.

Previous far-fault seismic monitoring data show that vertical seismic acceleration is less than horizontal^8^. Castelli et al.^9^ analyzed the interaction between soil and structure under vertical earthquakes. Button et al.^10^ and Wang et al.^11^ thought vertical seismic design is less important. With the improvement of monitoring level, more and more data show that the near-field vertical seismic acceleration amplitude is close to or even far beyond the horizontal seismic acceleration amplitude. The peak value of vertical and horizontal acceleration V/H of the Kobe earthquake in 1995 is close to 2^12^.

Thomas et al.^13^ conducted modal analysis on Tacoma bridge and found that ignoring vertical seismic component may bring greater risks, especially when the vertical excitation frequency of the ground is close to the natural frequency of the bridge. Borislav et al.^14^ discussed the influence of long term (LD) motion and near fault (NF) ground motion on seismic performance of seismic pier through numerical simulation, and compared with the response of short term far-field (FF) motion. A fiber-based nonlinear finite element bridge pier model is developed to evaluate the damage potential of different types of ground motion. Ayman et al.^15^ studied the velocity impact effect of bridge pier by using the method of near-fault impulse motion, in order to better understand the vibration dynamic behavior under forced vibration. Unlike foreign Bridges, most girder Bridges in China adopt rubber bearings and lack tensile strength^16,17^. The near-fault vertical earthquake may cause the main girder and bearing separation. To calculate vertical impact force, Yang et al.^4^ used the transient wave eigenfunction method to solve. However, Yang's study only considered the effect of separation on vertical impact but ignored the change of horizontal displacement of the bridge caused by separation. It is necessary to analyze the influence of the possible separation phenomenon on the damage of the horizontal bridge under the strong amplitude vertical earthquake motion.

Previous collision studies mainly focused on the longitudinal collision of adjacent beams^18–20^. Recently, Yang used the continuum model to calculate the vertical impact force between the main girder and the pier. However, the above study only considers the vertical earthquake and ignores the influence of the vertical separation of piers and beams on the longitudinal dynamic response of piers.

In this study, the influence of pier-beam separation on bridge force and displacement response are calculated by establishing a reliable theoretical method for dynamic bridge response. By using the mode superposition method, calculate the limit solution of the vertical impact force of the pier beam and the longitudinal deformation of the pier top after the first pier beam separation. The influence of pier beam separation on the pier failure model under different excitation frequencies was analyzed by calculating the vertical contact force of the pier beam and pier top offset.

## Theoretical model and vertical displacement calculation

### Theoretical model

**Figure 1:**
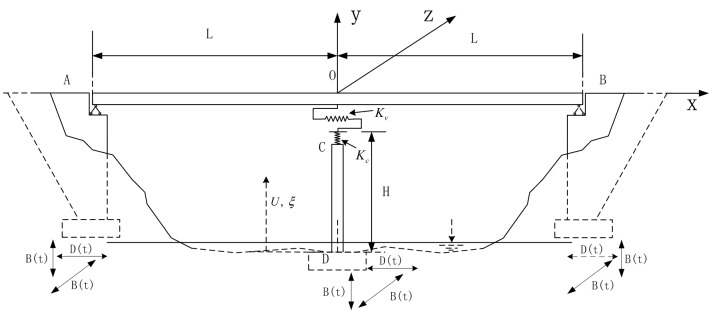
Bridge calculation model.

The calculation model selected in this study is a double-span continuous girder bridge. The calculation model is shown in Fig. 1. The main girder is a prestressed box girder, and both ends are articulated with the abutment. The bridge pier is double column round pier, the bottom and the foundation are just connected. The main beam and bridge pier are connected with plate rubber bearings. In the vertical and horizontal directions, the hysteresis curve of the support is long and narrow, and the damping of the support is ignored. In order to simplify the calculation of this study, the following assumptions are adopted in this paper:

1. When the bridge is forced to resonate, the structural force and displacement response are always calculated by elastic deformation.
2. Ignore the possible bearing shear failure caused by a horizontal earthquake.
3. Ignore the difference in the arrival time of the horizontal and vertical seismic waves, assuming that the three direction earthquakes are excited simultaneously.
4. It is assumed that the bridge is rigidly connected to the ground, and the coupling effect of soil and foundation is ignored.

### Theoretical solution of displacement response of bridge in longitudinal contact stage

Longitudinal displacement field of the girder can be divided into static displacement, rigid body displacement and dynamic deformation.

1 $$\begin{array}{*{20}c} \begin{gathered} X_{1} (x,t) = X_{1s} (x) + X_{1g} (x,t) + X_{1d} (x,t) \hfill \\ X_{2} (x,t) = X_{2s} (x) + X_{2g} (x,t) + X_{2d} (x,t) \hfill \\ \end{gathered} \\ {W(\xi ,t) = W_{s} (\xi ) + W_{g} (\xi ,t) + W_{d} (\xi ,t)} \\ \end{array}$$

X is the displacement of the girder and W is the displacement of the pier. The subscripts s, g and d are static displacement rigid body displacement and dynamic displacement respectively.

Static displacement and rigid body displacement of the bridge are as follows:

2 $$X_{1s} (x) = X_{2s} (x) = W_{s} (\xi ) = 0$$

3 $$X_{{{\text{1g}}}} (x,t) = X_{{{\text{2g}}}} (x,t) = W_{g} (\xi ,t) = D(t)$$

The dynamic deformation part of the structure are as follows:

4 $$\begin{aligned} X_{1d} (x,t) & = \sum\limits_{n = 1}^{\infty } {\varphi_{nb1} (x)q_{n} (t),} \quad X_{{{2}d}} (x,t) = \sum\limits_{n = 1}^{\infty } {\varphi_{{nb{2}}} (x)q_{n} (t)} \\ W_{d} (\xi ,t) & = \sum\limits_{n = 1}^{\infty } {\varphi_{nr} (\xi )q_{n} (t)} \end{aligned}$$

The equation includes the bending wave function ${\varphi }_{nb1},{\varphi }_{nb2}$ of the girder, the longitudinal wave function ${\varphi }_{nr}$ of the pier, and the time function ${q}_{n}(t)$.

By calculating boundary conditions and continuity conditions, obtain the wave functions of the bridge:

5 $$\begin{array}{*{20}c} {\varphi_{nb1} (x) = A_{n1} \sin k_{bn} x + A_{n1} \tan k_{bn} L\cos k_{bn} x} \\ {\varphi_{nb2} (x) = - A_{n1} \sin k_{bn} x + A_{n1} \tan K_{bn} L\cos k_{bn} x} \\ {\varphi_{nr} (\xi ) = M_{n1} (\sin k_{rn} \xi - \sinh k_{rn} \xi ) + M_{n1} E_{n1} (\cos k_{rn} \xi - \cosh k_{rn} \xi )} \\ \end{array}$$

where ${A}_{n1}$, ${M}_{n1}$ and ${E}_{n1}$ are the wave function coefficients.

The time function of the structure can be obtained by orthogonal consistency:

6 $$\omega_{n}^{2} q_{n} (t) + 2\zeta \omega_{n} \dot{q}_{n} (t) + \ddot{q}_{n} (t) = \ddot{Q}_{n} (t)$$

By Laplace transformation, ${q}_{n}(t)$ can be obtained:

7 $$\begin{aligned} q_{n} (t) & = e^{{ - \zeta \omega_{n} t}} \left( {q_{n} (0)\cos \omega_{d} t + \frac{{\dot{q}_{n} (0) + \zeta \omega_{n} q_{n} (0)}}{{\omega_{d} }}\sin \omega_{d} t} \right) \\ & \quad + \frac{1}{{\omega_{d} }}\int_{{0}}^{t} {e^{{ - \zeta \omega_{n} \tau }} \ddot{Q}_{n} (\tau )\sin (\omega_{d} (t - \tau ))d\tau } \\ \end{aligned}$$

where $\zeta$ is material damping. In Eq. (7), ${ \omega }_{d}=\sqrt{1-{\zeta }^{2}}{\omega }_{n}$.

In the separation stage, the girder and pier move at their respective frequencies, the classification of displacement response is consistent with the contact stage.

8 $$\begin{array}{*{20}c} {\overline{X} (x,t) = \overline{X}_{s} (x) + \overline{X}_{g} (x,t) + \overline{X}_{d} (x,t)} \\ {\overline{W} (\xi ,t) = \overline{W}_{s} (\xi ,t) + \overline{W}_{g} (\xi ,t) + \overline{W}_{d} (\xi ,t)} \\ \end{array}$$

The static displacement and rigid body displacement are consistent with the contact stage.

The calculation process of wave function in the separation stage is the same as that in the contact stage. During the calculation time, the structure may be separated several times. In that case ${t}^{*}=t-{t}_{2k}$ is the collision stage, ${t}^{*}=t-{t}_{2k+1}$ is the separation stage.

In the k-th separation process, the dynamic displacement responses of the main girder and pier are as follows:

9 $$\begin{aligned} q_{nb} (t^{*} ) & = e^{{ - \zeta \omega_{b1} t^{*} }} \left( {q_{1b} (t_{2k + 1}^{ + } )\cos \omega_{b1} t^{*} + \frac{{\dot{q}_{n} (t_{2k + 1}^{ - } ) + \zeta \omega_{b1} q_{nb} (0)}}{{\omega_{b1} }}\sin \omega_{b1} t^{*} } \right) \\ & \quad + \frac{1}{{\omega_{bd} }}\int_{{t_{2k + 1}^{ + } }}^{{t^{*} }} {e^{{ - \zeta \omega_{bn} \tau }} \ddot{Q}_{bn} (\tau )\sin (\omega_{bd} (t^{*} - \tau ))d\tau } \\ q_{nr} (t^{*} ) & = e^{{ - \zeta \omega_{r1} t^{*} }} \left( {q_{1r} (t_{2k + 1}^{ + } )\cos \omega_{b1} t^{*} + \frac{{\dot{q}_{n} (t_{2k + 1}^{ - } ) + \zeta \omega_{r1} q_{nr} (0)}}{{\omega_{r1} }}\sin \omega_{r1} t^{*} } \right) \\ & \quad + \frac{1}{{\omega_{rd} }}\int_{{t_{2k + 1}^{ + } }}^{{t^{*} }} {e^{{ - \zeta \omega_{rn} \tau }} \ddot{Q}_{rn} (\tau )\sin (\omega_{rd} (t^{*} - \tau ))d\tau } \\ \end{aligned}$$

When the vertical relative displacement between the middle of the main beam and the top of the pier is less than zero, the structure contacts again. In the collision stage, the dynamic response of the girder and pier is the displacement response of the separation stage superimposed by the collision displacement response. The indirect mode superposition method is adopted to calculate the structural displacement response caused by collision^21^, and the specific formula are as follows:

10 $$\begin{gathered} X_{F} (x,t) = \sum\limits_{n = 1}^{\infty } {\overline{\varphi }_{nb} (x)\left\{ \begin{gathered} e^{{ - \zeta \omega_{b1} t^{*} }} q_{nb} (t_{2k} )\cos \omega_{nb} (t - t_{2k} ) \hfill \\ + e^{{ - \zeta \omega_{b1} t^{*} }} \frac{{\dot{q}_{nb} (t_{2k} )}}{{\omega_{nb} }}\sin \omega_{nb} (t - t_{2k} ) \hfill \\ \int_{{t_{2k} }}^{t} {e^{{ - \zeta \omega_{rb} \tau }} \ddot{Q}_{nb} h_{nb} d\tau } \hfill \\ \end{gathered} \right\}} \hfill \\ W_{F} (\xi ,t) = \sum\limits_{n = 1}^{\infty } {\overline{\varphi }_{nr} (\xi )\left\{ \begin{gathered} e^{{ - \zeta \omega_{r1} t^{*} }} q_{nr} (t_{2k} )\cos \omega_{nr} (t - t_{2k} ) \hfill \\ + e^{{ - \zeta \omega_{r1} t^{*} }} \frac{{\dot{q}_{nr} (t_{2k} )}}{{\omega_{nr} }}\sin \omega_{nr} (t - t_{2k} ) \hfill \\ \int_{{t_{2k} }}^{t} {e^{{ - \zeta \omega_{rn} \tau }} \ddot{Q}_{nr} h_{nr} d\tau } \hfill \\ \end{gathered} \right\}} \hfill \\ \end{gathered}$$

where ${Q}_{nb}, {Q}_{nr}$ are the generalized collision forces. ${x}_{0}$ and ${\xi }_{0}$ are the coordinate of the collision points of the main beam and pier, respectively. ${h}_{nb}$ and ${h}_{nr}$ are the impact impulse response functions.

## Numerical simulation and analysis

**Figure 2:**
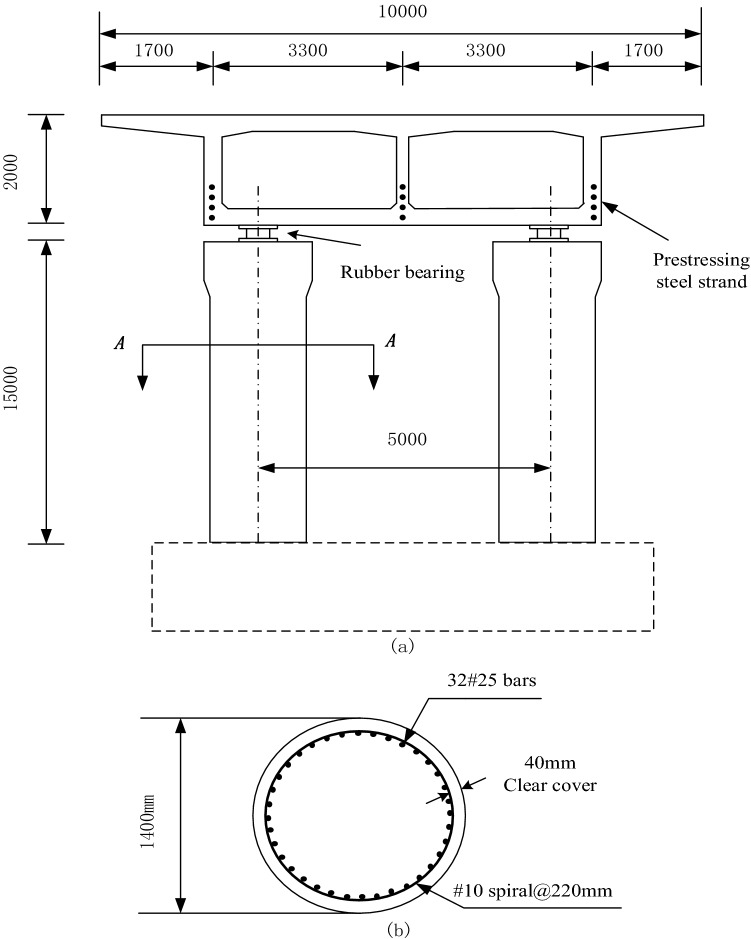
Dimensions and details of the bridge elevation and section drawings.

In this paper, a two-span prestressed continuous girder bridge in China is selected. See Fig. 2 for the specific section. Considering that the damping of the rubber bearing is very small, the hysteretic curve of the material is long and narrow, the structural damping is ignored. The rubber bearing is simulated by two springs, axial stiffness ${K}_{c}=2.4\times {10}^{9}\,{\text{N/m}}$, and shear stiffness ${K}_{v}=2.4\times {10}^{6}\,{\text{N/m}}$. The flexural vibration of the bridge, the damping coefficients of the main girder and pier are assumed to be ${\zeta }_{2}$ = 2%.

### Influence of structural separation on horizontal displacement of bridges

Since the natural period of the bridge is inconsistent in the vertical and horizontal directions, the seismic excitation period selected in this paper is close to the horizontal and vertical natural periods for analysis. To ensure the accuracy of the calculation, In addition, to avoid the complexity of calculation, the appropriate time-step increments must be selected. For the selection of time-step increments, it is necessary to clearly express the characteristics transmitted in the girder and pier, so a small time-step increase is required for analysis during calculation. The longitudinal wave velocity of the bridge pier is ${c}_{r}=\sqrt{{E}_{r}/{\rho }_{r}}$ = 3492 m/s, and the bending wave velocity is ${a}_{r}=\sqrt{{E}_{r}{I}_{r}/{\rho }_{r}{A}_{r}}$=1060 m/s. The maximum time-step increments must be less than the time for the bending wave and axial wave traveling across the entire pier, $\Delta t<L/{c}_{r}$ = 4.3e−3 s. Hence, time-step increments 1.0e−3 s are used for research. Another parameter to be considered in this study is the modal cutoff number of the wave. Considering the influence of damping on high-frequency vibration, the number of modes selected in this study is N = 5.

Considering the separation condition, the horizontal displacement of the bridge is affected by the vertical displacement. Put the recorded time into the calculation of horizontal displacement, and obtain the horizontal displacement of the bridge under the condition of considering separation. The total computation time is 2 s.

**Figure 3:**
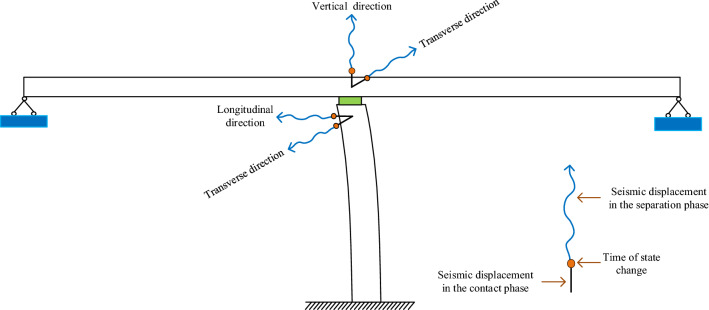
Diagram of displacement response of bridge under multiple structural separations.

Figure 3 shows the model diagram of the seismic response of the bridge considering the separation condition. The straight line represents the displacement response in the contact stage, the origin represents the point of state change, and the curve represents the displacement response in the separation stage. Therefore, in the horizontal direction, when a vertical earthquake separates the main girder and pier, the structure is in a contact–separation–recontact state. Meeting and always in contact, separation may affect horizontal displacement.

**Figure 4:**
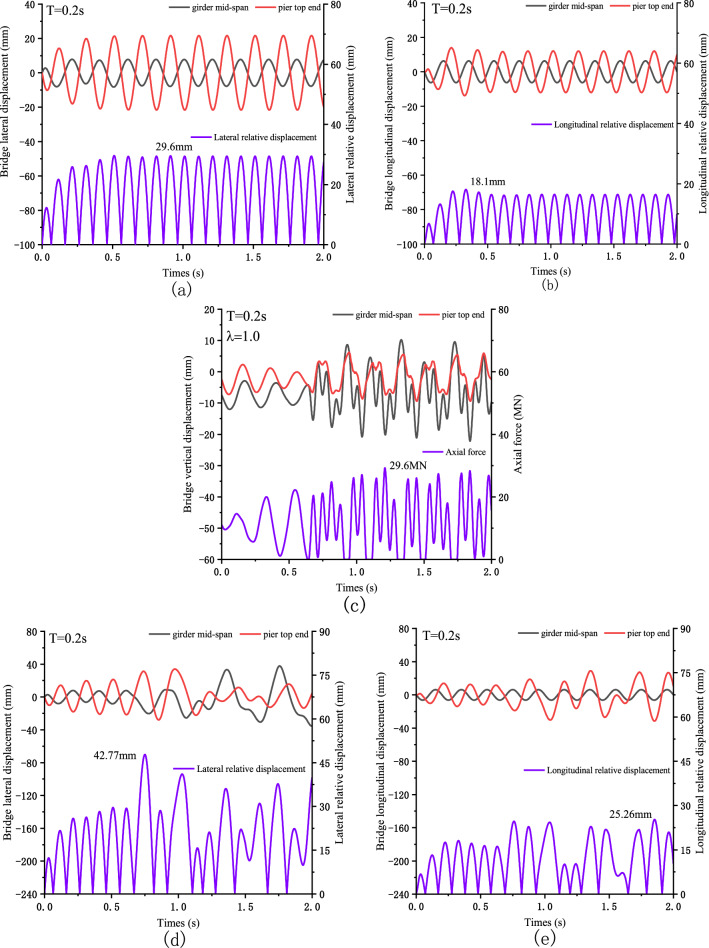
Seismic displacement response of Bridges: (**a**) lateral direction is not separated; (**b**) longitudinal direction is not separated; (**c**) vertical displacement; (**d**) lateral separation occurs; (**e**) longitudinal separation occurs.

Figure 4 shows the seismic response of the bridge when T = 0.2 s. Figure 4a,b shows the horizontal displacement of the bridge under the condition of ignoring separation. In the lateral direction, the maximum relative displacement of the main girder and pier is 29.6 mm. In the longitudinal direction, the maximum relative displacement is 18.1 mm. However, in the vertical direction, the main girder and pier separation will occur when T = 0.2 s. Figure 4c shows that the bridge is separated six times in 2 s, and the maximum vertical impact force generated is 29.6 MN, which is 2.47 times the static force. Figure 4d,e shows the horizontal displacement of the bridge with separation considered.

By comparing Fig. 4a,b, it can be seen that vertical separation greatly influences horizontal displacement response. Under the separation condition, the maximum lateral relative displacement increases from 29.6 to 42.77 mm, an increase of 44.5%. The maximum relative displacement increases from 18.1 to 25.26 mm in the longitudinal direction, increasing by 39.6%. In the horizontal direction, the maximum displacement in the lateral direction is greater than that in the longitudinal direction. This may be because the main girder and bridge pier have greater flexibility in the horizontal direction, while the main girder has greater stiffness in the longitudinal direction. Therefore, the effect of the possible separation on the structure should be considered in the near-fault earthquake.

### Effect of separation on eccentric compression

**Figure 5:**
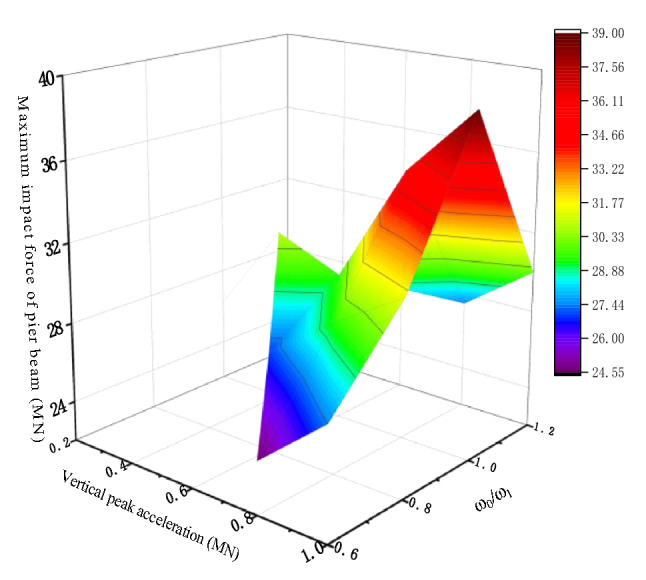
The vertical impact force of pier beam.

Figure 5 shows the vertical impact force of the pier beam under different vertical excitation peak acceleration and excitation frequency. The results show that the collision mainly occurs when the excitation frequency is close to the natural vertical frequency of the bridge, and the closer the frequency is to the vertical, the greater the collision force is. In this study, the effect of vertical separation of pier and beam on pier failure is mainly considered, and the seismic excitation period selected is T = 0.2 s.

In the analysis of the above model, the following assumptions are made for the convenience of calculation:

1. Ignoring the time difference between vertical earthquake and horizontal earthquake, set it as simultaneous excitation.
2. The elastic model is always used to calculate the displacement response of the structure, and the plastic deformation is ignored.
3. When calculating the bending moment caused by eccentric impact, the coupling effect of pier vibration and bearing shear is ignored.
4. For the possible second-order effect of the bridge pier, the method coefficient $\eta$ in the specification is substituted into the calculation.

**Figure 6:**
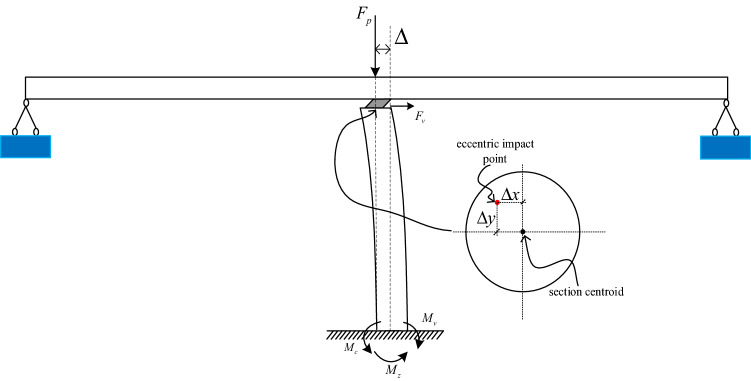
Schematic diagram of the bending moment at the bottom of the pier.

**Figure 7:**
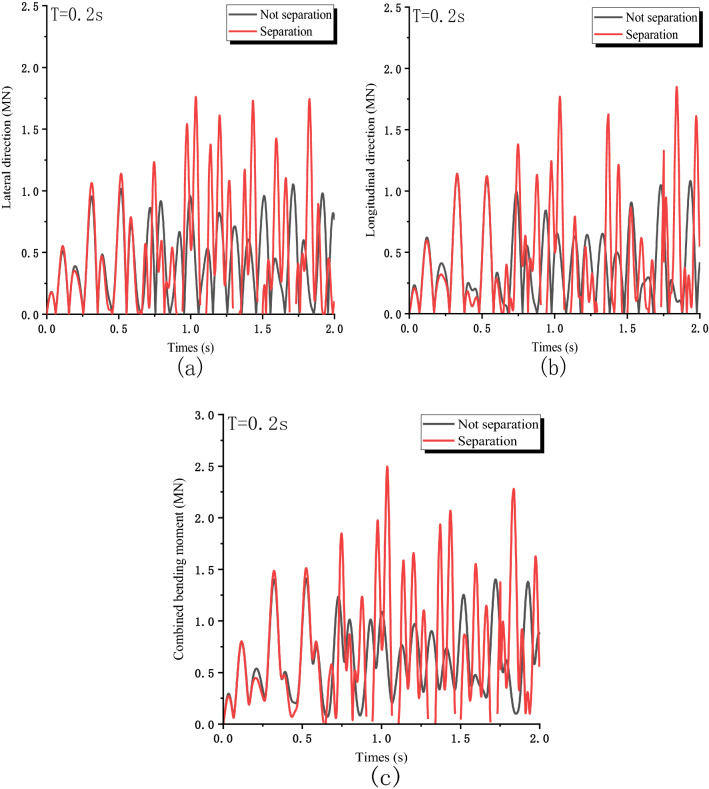
The bending moment generated by eccentric compression at the bottom of the pier: (**a**) Lateral direction; (**b**) Longitudinal direction; (**c**) Horizontal bending moment.

The collision diagram is shown in Fig. 6. The vertical collision force can be calculated ${\mathrm{M}}_{\mathrm{c}} = F\times\upeta \times \Delta$. In the lateral direction $\Delta$ is the lateral impact eccentricity and in the longitudinal direction $\Delta$ is the longitudinal impact eccentricity, $\upeta$ is the eccentricity amplification factor. Figure 7 shows the bending moment generated by eccentric compression at the bottom of the pier. In the lateral direction, the bending moment caused by eccentric impact increases from 1.05, to 1.76 MN m. The longitudinal direction increased from 1.13 to 1.85 MN m. In the horizontal direction, the bending moment caused by eccentric impact increases from 1.41 to 2.5 MN m. Therefore, the eccentric collision caused by separation may increase the bending moment of the pier or even cause the pier failure.

**Figure 8:**
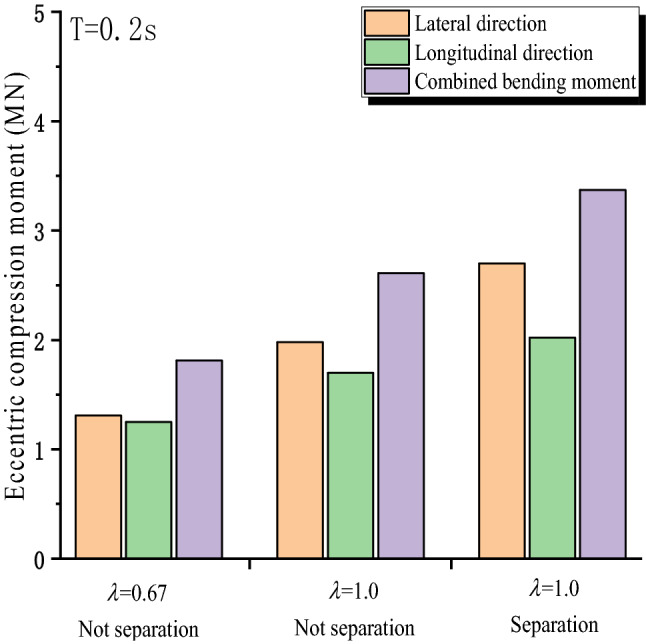
The bending moment caused by eccentric compression under the most unfavorable conditions.

Figure 8 shows the variation of horizontal bending moment of bridge piers under three conditions. Ignored the separation of pier and beam caused by vertical seismic excitation, with the increase of $\lambda$(V/H) amplitude; the combined bending moment goes from 1.81 to 2.6 MN m by 43.7%. The increase of vertical force will enlarge the eccentric impact moment. When structural separation is considered, the expanded deformation of 0th pier roof also increases eccentricity, the bending moment increases from 2.61 to 3.4 MN m. Therefore, when considering vertical seismic action, it is necessary to consider the vertical force and the separation that may occur.

### Influence of pier length on structural failure

**Figure 9:**
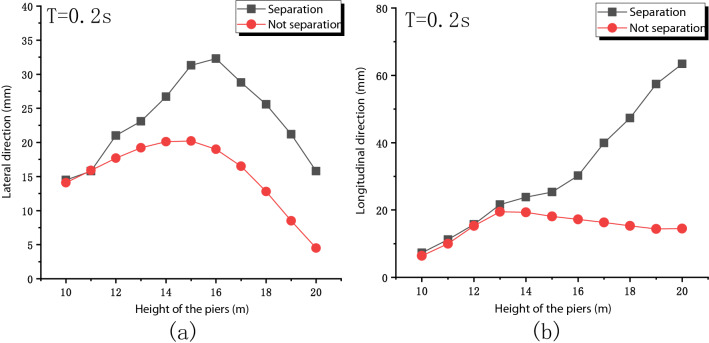
Horizontal deformation of pier top under different pier heights: (**a**) Lateral direction; (**b**) Longitudinal direction.

Figure 9 shows the horizontal deformation of the pier top under different pier heights. The separation will increase the deformation at the top of the pier in both the lateral and longitudinal directions. But the trends in the two directions are different. In the lateral direction, the deformation of the pier top increases first and then decreases. In contrast, in the longitudinal direction, it increases monotonically. The reason for this situation is that the lateral natural vibration period of the bridge is affected by the main girder, while the longitudinal natural vibration period of the bridge is mainly affected by the pier. At the peak point of relative transverse displacement, the period of natural vibration of the bridge pier is close to the period of natural vibration of the bridge. In the longitudinal direction, the period of natural vibration of the pier is close to that of the bridge. The relative displacement increases with the increase of the pier height.

### Influence of girder span on structural failure

**Figure 10:**
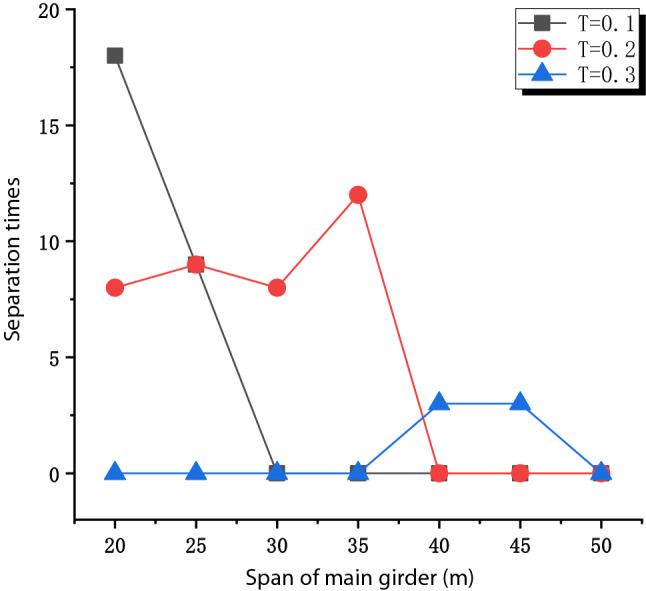
Bridge separation times under different girder spans.

Figure 10 shows the separation times of Bridges with different main girder spans. The seismic excitation periods given are T = 0.1 s, T = 0.2 s, and T = 0.3 s. The bridge separation occurs only when the seismic excitation period is close to the vertical natural vibration period. With the increase of the span of the main girder, the separation interval becomes narrower, and the number of separations becomes less and less. This is because the amplitude of V/H is higher in the short period interval and lower in the long period interval under the action of near-fault vertical earthquakes.

**Figure 11:**
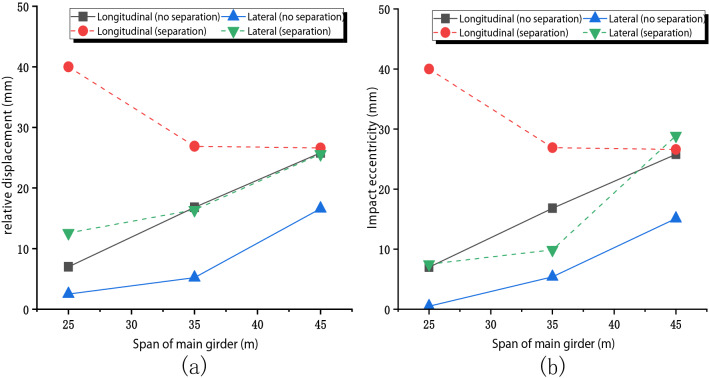
Influence of separation on the bridge under different main girder span: (**a**) Relative displacement; (**b**) Impact eccentric.

To analyze the horizontal displacement of the bridge under different main girder spans, the seismic excitation period was chosen as T = 0.2 s. The main girder span of the three bridges is 25 m, 35 m, and 45 m (all the three bridges will separate when T = 0.2 s). Figure 11 shows the horizontal seismic response of the bridge with different spans. Laterally, with the increase of beam span, the influence of separation on the relative displacement of pier beam decreases gradually. When L = 45 m, the influence of separation on the relative displacement of the pier girder is very small. In the longitudinal direction, the separation increases the relative displacement of pier and beam regardless of the main girder size.

Eccentric distance determines the bending moment generated by the eccentric impact on a bridge pier. In the longitudinal direction, with the span increase, whether the bridge is separated shows different trends and is nearly equal at last. In the lateral direction, the eccentricity increases monotonically with the increase of span. When the pier height is low, the eccentric impact mainly comes from the longitudinal direction. With the increase of the pier height, the influence of eccentric impact in the lateral direction gradually increases, and even exceeds the longitudinal direction.

### Finite element analysis

The previous research calculates the bending of the pier under separation conditions through a theoretical solution. However, the coupling effect between vertical and horizontal is ignored in the theoretical solution. To verify the correctness of the theory, ANSYS modeling is used for comparative analysis.

The two ends of the main beam are hinged points with three-dimensional constraints, and the bottom of the pier is a rigid node. BEAM 188 unit is adopted for the main beam and pier. For bridge bearings, different elements are used in the vertical and longitudinal directions. The vertical direction is set as LINK10 element, and the bearing height is ∆Z when not stressed. In the longitudinal direction, the COMBIN 14 element is adopted, and the spring stiffness is the shear stiffness of the support. The bearing is bonded with the pier and overlapped with the main beam.

**Figure 12:**
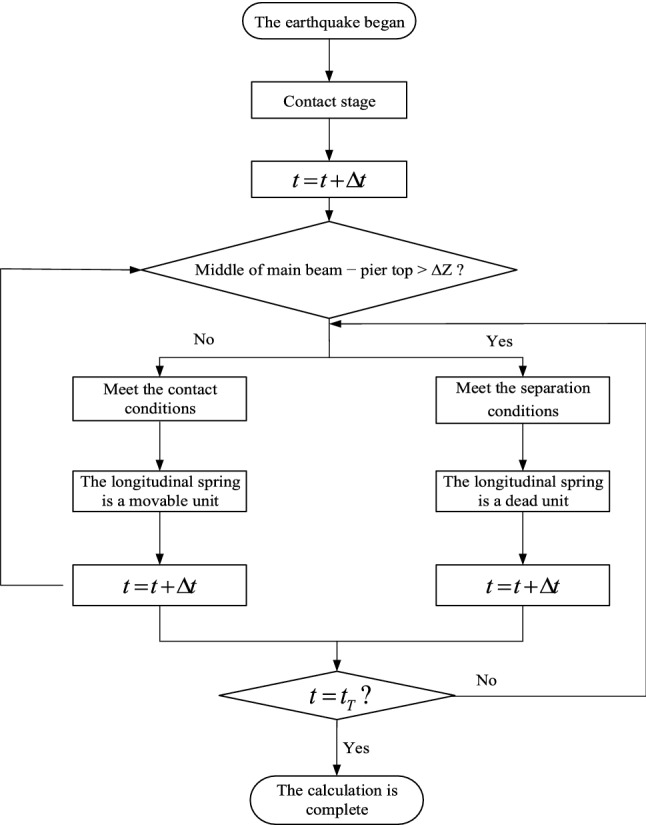
Flow chart of finite element calculation.

To simulate the pier-beam separation condition caused by near-fault vertical earthquake excitation, when the vertical relative displacement of the middle of the main beam and the top of the pier is greater than the support height ∆Z and shows an increasing trend, it means that the main beam and the pier are separated in the vertical direction. The longitudinal spring element is set as a dead element, and the main beam and the pier are not connected in the vertical direction. When the vertical relative displacement between the middle of the main beam and the top of the pier is less than the support height ∆Z and shows a decreasing trend, the main beam, and the pier collide vertically, the longitudinal spring element is a live element, and the main beam and the pier are connected. See Fig. 12 for a specific calculation.

**Figure 13:**
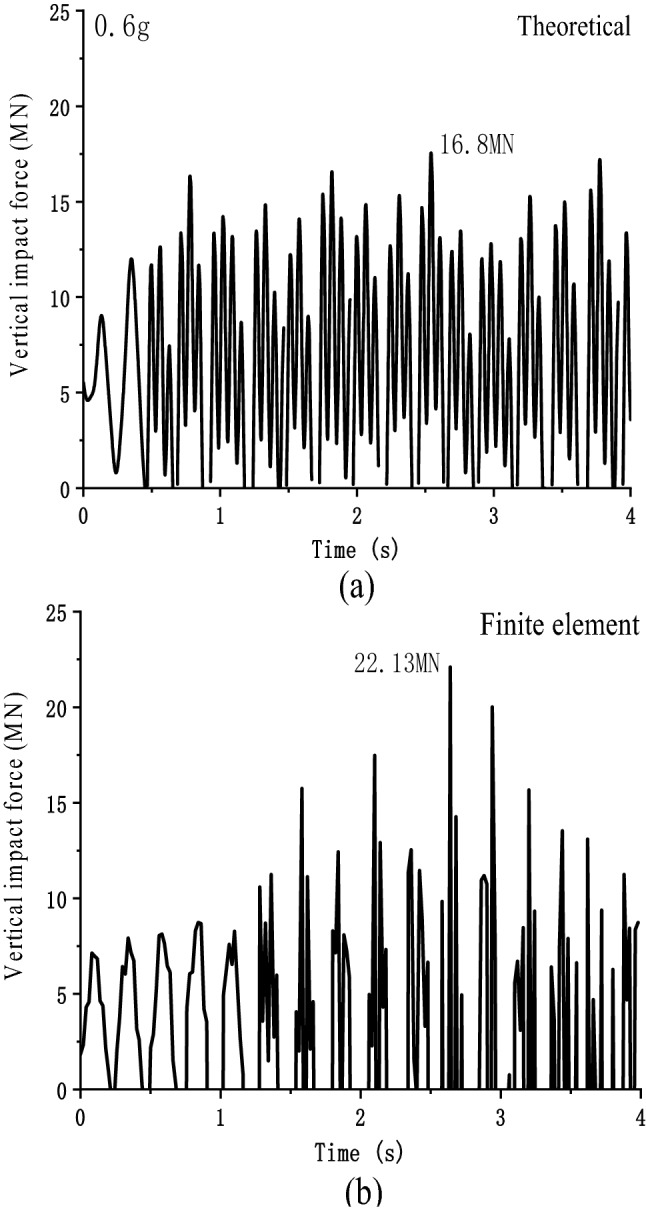
Vertical axial pressure of pier: (**a**) Theoretical solution; (**b**) Finite element solution.

The main beam and pier are separated when the seismic excitation period T = 0.25 s and the vertical seismic peak acceleration is 0.6 g. Figure 13 shows the axial pressure of the pier under the finite element solution and theoretical solution. Under the theoretical solution, the maximum axial pressure on the pier is 16.8 MN, 2.8 times the static pressure. Under the finite element solution, the maximum axial pressure on the pier is 22.13 MN, 3.67 times the static pressure. The finite element solution is slightly larger than the theoretical solution.

**Figure 14:**
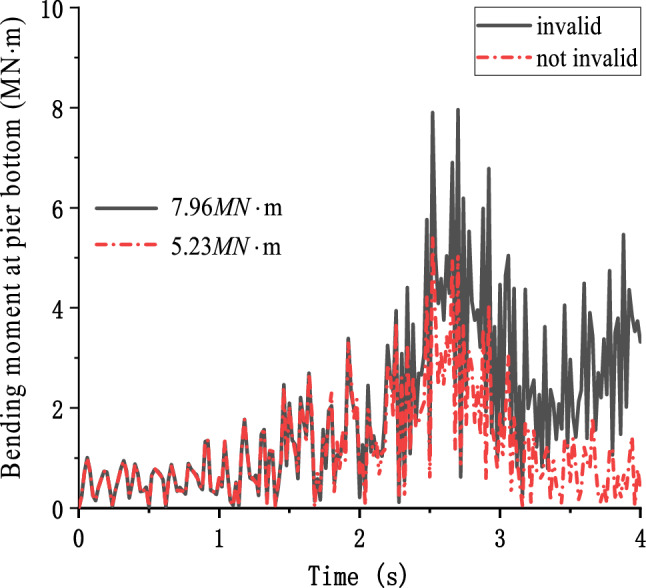
Bending moment at pier bottom.

Due to the large impact force on the pier and the change of longitudinal deformation caused by vertical seismic excitation, the bending moment at the bottom of the pier increases significantly. At the same time, when considering the failure of longitudinal support restraint when the bridge separation is considered, the maximum bending moment at the bottom of the pier is 7.96 MN m, which is greater than 5.23 MN m when the failure of longitudinal restraint is ignored, which is 47.05% higher (Fig. 14). When the theoretical solution is adopted, and the longitudinal separation is ignored, the maximum bending moment at the bottom of the pier under the most unfavorable condition is 3.46 MN m. When considering separation, the maximum bending moment at the bottom of the pier is 6.74 MN m.

Comparing the theoretical solution with the finite element solution, the following conclusions can be summarized: (1) with the increase of the amplitude of vertical seismic acceleration, the bending moment at the bottom of the pier also increases; (2) when the vertical excitation amplitude is large, ignoring the coupling effect has the risk of underestimating the bending of piers; (3) the vertical separation of the structure will increase the bending moment at the bottom of the pier and increase the risk of bending failure of the pier; (4) with the increase of vibration duration, the bending moment at the bottom of pier increases gradually. At the same time, the influence of separation on the flexural failure of piers is mainly concentrated in the section with large moment fluctuation. At this time, the pier amplitude is large; (5) when using finite element calculation, the axial force increases greatly after a small fluctuation, and the bending moment at the bottom of the pier also increases greatly.

## Conclusions

This paper establishes a girder–spring–pier model to analyze the possible changes of main girders and piers under near-fault earthquake conditions. The influence of eccentric vertical impact on the pier is calculated by analyzing the deformation of the top of the pier caused by separation. By calculating the response of bridges of different sizes under earthquake, the following conclusions are drawn:

1. Under near-fault earthquake, when the seismic excitation period is close to the vertical natural period of the bridge, the vertical separation of pier and beam may be caused. The larger the natural vibration period, the less the separation times and the smaller the separation interval.
2. With the increase of V/H amplitude, the increase of bending moment caused by eccentric impact is not only from the increase of vertical contact force, but also from the increase of transverse and longitudinal deformation at the top of the pier.
3. With the increase of pier height, the excitation acceleration required for pier and beam separation increases. The separation of piers and beams will affect the horizontal dynamic response of Bridges. In the transverse direction, the maximum deformation of pier top increases first and then decreases with the increase of pier height. In the longitudinal direction, the maximum deformation of pier crest increases monotonically.
4. With the increase of girder span, the influence of separation on the longitudinal deformation of the pier top is not consistent. However, in the lateral direction, the maximum deformation increases with the increase of girder span regardless of separation.
5. When the vertical excitation amplitude is large and the pier height is high, ignoring the horizontal and vertical coupling effect may underestimate the risk of pier bending.
